# Intravesical botox as an effective therapy for giggle incontinence in children

**DOI:** 10.1007/s00383-026-06297-7

**Published:** 2026-02-18

**Authors:** Eliza Szwarcberg, Chris Kimber, Kiarash Taghavi

**Affiliations:** 1 Department of Paediatric Urology, Monash Children’s Hospital, 246 Clayton Rd, Melbourne, Australia; 2 Department of Paediatrics, School of Clinical Sciences, Faculty of Medicine, Nursing and Health Sciences, Monash University, Melbourne, Australia

**Keywords:** Giggle incontinence, Urinary incontinence, Botulinum toxins, Type A, Paediatrics

## Abstract

**Purpose:**

Giggle incontinence is a bladder storage disorder characterized by uncontrolled voiding during or immediately after laughter. Many children are refractory to available therapies or experience considerable side-effects. This study aimed to determine the efficacy of intravesical Botox injections in the management of giggle incontinence.

**Methods:**

A retrospective review of all children who received 200iU intravesical *botulinum toxin-A* for giggle incontinence over twelve years was performed. All children experienced complete bladder emptying during or immediately after laughter as their primary complaint, with or without a related voiding disorder. Outcomes were characterised as: “no response” (0–49% reduction), “partial response” (50–99% reduction), or “complete response” (100% reduction).

**Results:**

A total 34 procedures (range: 1–5) in 17 children were included. Median age at first treatment was 11 years (range: 6-17y). Complete response occurred in 65% of patients and partial response in 18%. Of those who experienced complete response, 55% (6/11) had no relapse of symptoms with a median duration of follow-up of 5 years (IQR 3.5-6.5y).

**Conclusion:**

Giggle incontinence is a rare but significant condition with marked impact on quality of life. This is the first study describing the use of intravesical Botox injection in this population, demonstrating safety and efficacy with a sustained response to treatment in many patients.

**Supplementary Information:**

The online version contains supplementary material available at 10.1007/s00383-026-06297-7.

## Introduction

Giggle incontinence is a bladder storage disorder characterized by uncontrolled voiding during or immediately after laughter. The exact pathophysiology of giggle incontinence is unclear, though it is likely to be a combination of lower urinary tract dysfunction and neurologically-mediated factors. Suggested management strategies generally target one of two levels: supratentorial (methylphenidate) or lower urinary tract (urotherapy, oxybutynin, biofeedback). However, at present management is guided by small case series, and there is no consensus regarding optimal management [1]. Many children with giggle incontinence do not respond to the currently available interventions or experience significant side-effects as a result.

Onabotulinum toxin A (Botox, Allergan, Irvine, CA, USA) is commonly used as an intravesical injection in children with other refractory functional bladder disorders to achieve neuromuscular stabilisation of the detrusor and improve functional capacity. To date, there is a single case report of one adult with giggle incontinence receiving intravesical Botox injection and it’s use in children for this indication has not previously been reported [2]. Nonetheless, the safety of intravesical Botox has been well established in children of all ages with functional urinary incontinence [3]. The present study aimed to determine the efficacy of intravesical Botox injections in the management of children with giggle incontinence.

## Materials and methods

The medical records of all children who underwent intravesical Botox injection by multiple surgeons for management of giggle incontinence between January 2013 and December 2024 were retrospectively analysed. All children included in this study experienced complete bladder emptying during or immediately after laughter as their primary presenting complaint, with or without the presence of a related voiding disorder.

Invasive cystometrography was not performed pre-operatively due to the invasive nature of this test. Pre-operative uroflowmetry and renal tract ultrasound (with pre- and post-void bladder volumes) were performed on a case-by-case basis, but treatment decisions were made predominantly based on clinical grounds and non-invasive urodynamic studies. No child had significant post-void residual volumes pre-operatively.

All children received 200iU of botulinum toxin A (Botox, Allergan, Irvine, CA, USA). Rigid cystoscopy was performed with a Storz Paediatric Cystoscope (9.5Fr). A Williams Cystoscopic Injection Needle (Cook Medical) was utilised for intra-muscular injection, which has a 23G needle tip, and 8mm tip.

The product was diluted into 20mL of normal saline with a sterile technique, and injected in twenty 1mL aliquots. The technique utilised intramuscular (detrusor) injections (up to a 6 mm depth) commencing in the midline trigonal region and continuing over the base and dome of the bladder (avoiding the ureteric orifices and the ureteric intravesical course). All children received a 25 mg/kg dose of intravenous cephazolin at induction. The treatment was performed under general anaesthesia in all cases.

All children were routinely reviewed at 4 weeks post-operatively with subsequent ongoing follow-up arranged on a case-by-case basis depending on clinical course.

The severity of giggle incontinence was classified as mild (episodes of giggle incontinence occurring more than once monthly but less than twice per week), moderate (more than once per week but not daily), and severe (at least daily).

Treatment outcomes were characterised as “no response” (0–49% reduction in number of episodes of giggle incontinence), “partial response” (50–99% reduction) or “complete response” (100% reduction) as per the International Children’s Continence Society (ICCS) recommendations [4]. Treatment outcomes related specifically to reduction in frequency of episodes of giggle incontinence, i.e. complete bladder emptying during or immediately after laughter. Treatment outcome was assessed following the final treatment cycle when multiple treatments were undertaken on the same child.

## Results

During the study period, 19 children underwent cystoscopic intradetrusor injection for giggle incontinence and 17 children (10 males) who underwent a total of 34 injection procedures were included in the final analysis. Two children were excluded due to inadequate follow-up information. Median age at first treatment was 11 years (range 6–17 years).

Four children (29%) had isolated giggle incontinence with no other related voiding disorder. Concurrent symptoms of functional bladder dysfunction as defined by the ICCS are presented in Table 1. Co-morbidities included posterior urethral valves (*n* = 1), unilateral duplex kidney (*n* = 1), horseshoe kidney (*n* = 1), Crohn’s disease (*n* = 1) and infantile myofibromatosis (*n* = 1). No children had a documented co-existing neuropsychiatric condition. One child had a history of two afebrile urinary tract infections. One 14 year old girl who received five treatments in total, received 300iU for one injection procedure as there was a lesser clinical response noted as the child grew.

Past treatments included oxybutynin in 11 patients, specialised continence physiotherapy in 9 patients, mirabegron in 1 patient, tolterodine in 1 patient and methylphenidate in 1 patient. All children who experienced constipation (*n* = 3) were treated with aperients with nil or minimal effect on giggle incontinence. No child had previous intravesical Botox injection procedures prior to the study period.

Before treatment, 12 children (71%) experienced moderate giggle incontinence and five children (29%) experienced severe giggle incontinence. The mean number of treatment cycles required per patient during the study period was 2 (range 1–5, detailed in Table 2). One female was subsequently transitioned to adult urology for ongoing management of giggle incontinence.

**Table 1:** Concurrent symptoms of functional lower urinary tract dysfunction as per ICCS

Symptom	*n* (%)
Urgency	10 (59%)
Daytime incontinence at other times	6 (35%)
Bladder spasms	3 (18%)
Frequency	2 (12%)
Nocturnal enuresis	0 (0%)

**Table 2:** Number of treatment cycles performed during the study period breakdown of treatment response according to severity and associated symptoms are presented in Tables 3 and 4

Number of Treatment Cycles	*n* (%)
1	7 (41%)
2	7 (41%)
3	1 (6%)
4	0 (0%)
5	2 (12%)

**Table 3:** Treatment response according to severity of giggle incontinence prior to initial injection procedure

Pre-op severity	No response	Partial response	Complete response	
Moderate	3 (23%)	2 (15%)	8 (62%)	13
Severe	0	1 (25%)	3 (75%)	4
	3 (18%)	3 (18%)	11 (65%)	17

**Table 4:** Treatment response according to presence of a related voiding disorder

	No response	Partial response	Complete response	
No related voiding disorder	2 (50%)	0	2 (50%)	4
Presence of related voiding disorder	1 (8%)	3 (23%)	9 (69%)	13
	3 (18%)	3 (18%)	11 (65%)	17

Of those who experienced a complete response to treatment, 55% (6/11) had no further episodes of giggle incontinence with a median duration of follow-up of 5 years (IQR 3.5–6.5.5y). The median duration of response in those who experienced a relapse of symptoms was 12 months (IQR 6.5–12.5 months). The median time between treatment cycles in children who underwent multiple treatment cycles was 14 months (IQR 7–24 months).

There were no intra-operative complications recorded. Post-operative complications included post-operative abdominal pain in one girl aged 14 years following her second injection procedure, where 300iU of Botox was administered. The pain self-resolved after one month. There were no instances of post-operative urinary tract infection and no cases of urinary retention.

Four children (3 males) aged 7–13 years at first treatment had isolated giggle incontinence with no other related voiding disorder. One boy who had two treatment cycles with a 14 month interval. He achieved a complete response for 12 months following the repeat procedure. Another boy had two treatment cycles with a seven month interval. He reported complete response to the first procedure for five months, however had no response to the repeat procedure. One boy had one injection procedure, with posterior urethral valve resection at that time, and had complete response with relapse of symptoms after one month. One girl had a single treatment cycle with no response.

## Discussion

Giggle incontinence is a rare but significant condition that has marked impacts on children’s quality of life. There is no consensus regarding optimal management and many children are refractory to current treatment options, experience significant adverse effects, or have regression of symptoms after cessation [1, 5]. Intravesical Botox injection provides an alternate therapy for these children. The present study investigated efficacy and, to our knowledge, represents the first series of children undergoing intravesical Botox injection for this indication. It confirms the procedure as an effective, well-tolerated intervention in the management of children with giggle incontinence.

Giggle incontinence has historically been variably defined. The ICCS define giggle incontinence as extensive emptying or leakage which occurs during or immediately after laughing, with normal bladder function when there is no laughter [4]. The ICCS definition including “with normal bladder function when there is no laughter” is vague, and does not define the extent to which bladder function needs to be defined and investigated. For example, we believe it is inappropriate for all children who suffer from symptoms of giggle incontinence to undergo invasive urodynamic studies. Therefore we utilised a modified ICCS definition with a clinical emphasis: episodes of significant voiding occurring during or immediately after laugher, with or without the presence of a related voiding disorder. In the context of this study, it can be argued that this provides a more clinically useful definition which accounts for the multifactorial nature of giggle incontinence [6]. In the current study, 76% of children reported the additional episodes of bladder dysfunction unrelated to laughter, suggesting that a high proportion of patients present a mixed picture.

The aetiology and pathophysiology of giggle incontinence appears as a complex interaction incorporating both centrally-mediated neurological components and local dysfunction of the detrusor and pelvic floor muscles underlying the condition. A popular theory describes a neurological cascade triggered by laughter combined with detrusor instability resulting in bladder emptying [1, 7]. In support of this theory, previous studies have demonstrated a correlation between clinically isolated giggle incontinence and idiopathic detrusor overactivity on urodynamic studies [7–10]. Further, it has been shown that dysfunctional voiding as determined by uroflowmetry-EMG is a predictive factor for resistance to current treatments for giggle incontinence [10]. It appears that in these children laughter triggers asensate detrusor overactivity and concurrent momentary pelvic floor relaxation [7]. Here, intravesical Botox acts locally to address the end-organ component of giggle incontinence. It may be that in the partial or non-responders, either a therapeutic threshold was not obtained or there were other mechanisms explaining the cause of their giggle incontinence.

Intravesical Botox is a safe and effective treatment in the management of children of all ages with functional urinary incontinence [3]. In the context of giggle incontinence there is a single case report detailing intravesical Botox with complete and sustained symptom resolution [2]. In the current study, 82% (14/17) of patients experienced a partial or complete response to treatment, the majority of which had been refractory to alternative measures. Notably, 50% (2/4) of children who had no additional symptoms of bladder dysfunction reported complete response to treatment.

Complete response with no relapse two years after treatment occurred in 50% (5/10) of children. Given the natural history of giggle incontinence is unknown, it is difficult to determine whether the sustained resolution of symptoms is a response to treatment, or related to the natural history of the condition. From foundational knowledge of other functional bladder conditions, it is likely an interplay between response to treatment, plasticity of the system, and a new found physiological setpoint that results in sustained resolution.

The median duration of effect in those who experienced a relapse of symptoms after initial complete response was 12 months. Previous studies observing the effect of Botox in non-neurogenic detrusor overactivity have reported shorter median response durations, ranging from five to eight months [11–13]. Interestingly, Hoebeke et al. noted that eight of the nine full responders in the study were still cured after 12 months, and one had relapse after eight months [14]. 

The longer duration of effect observed in the present study may represent a type 1 error. However, it should be noted that the exact mechanism of Onabotulinum toxin A in this context remains poorly understood. While it is clear that Botox targets the end organ component of giggle incontinence, it is not known whether its therapeutic effect is mediated by the afferent or efferent pathways. Perhaps this complex interaction is responsible for the longer duration of effect in giggle incontinence, as compared to non-neurogenic detrusor overactivity. A final possibility for the increased duration of effect is that there may be a lower therapeutic threshold in this cohort.

It is challenging to compare the efficacy of intravesical Botox injections with alternative treatment options as studies are generally limited by small sample size due to the low prevalence of giggle incontinence. Nevertheless, standard urotherapy has shown a partial response in approximately one third of patients, and biofeedback has been reported as effective in 73% of patients [10, 15]. Methylphenidate has shown varying results, ranging from 55 to 100% efficacy [5, 9, 10, 16–19]. The wide variability is likely accounted for by small sample sizes as well as variations in dose, duration of treatment and duration of follow-up. Additional limitations of methylphenidate include relapse of symptoms after cessation of methylphenidate, continued wetting in the evening as an end dose effect, and intolerance of adverse effects (irritability, agitation, sleep disturbance, decreased appetite) [5, 17, 19]. 

There are several further limitations of the current study. The retrospective nature is a source of reporting or recall bias, particularly in those children who received multiple treatment cycles. To minimise recall bias, the study analysed only response after the final treatment in the study period. However, this introduces heterogeneity, with outcomes measured after varying numbers of injection procedures. The inclusion of children with or without a related voiding disorder also introduced heterogeneity. The case numbers were limited because of the relative rarity of the condition. This, in addition to risk of inter-operator variability, may limit the generalisability and reproducibility of the study. The study is also limited by a lack of control data. These limitations may be overcome by multi-institutional prospective trials comparing treatments, and analysing outcomes after both initial and subsequent injection procedures. However such a study has practical challenges to complete.

## Conclusions

Intravesical Botox injection is an effective and well-tolerated treatment option for children with giggle incontinence. This is the first study describing its’ use in this population, demonstrating safety and efficacy with a sustained response to treatment. Use of intravesical Botox should be considered in giggle incontinence, particularly given the reported profile of alternative therapies. Further studies are needed to better define its efficacy and confirm its role in the management of giggle incontinence, in those with and without co-existent voiding disorders.

## Supplementary Information

Below is the link to the electronic supplementary material.

Supplementary Material 1

## Data Availability

The data sets generated during and/or analysed during the current study are available from the corresponding author on reasonable request.

## Declarations

of interest: none.
